# Ankle fusion with centralisation of the fibula after distal tibia bone tumour resection

**DOI:** 10.1007/s10195-013-0279-2

**Published:** 2013-12-15

**Authors:** Zile Singh Kundu, Paritosh Gogna, Vinay Gupta, Rohit Singla, Sukhbir Singh Sangwan, Mukul Mohindra, Amanpreet Singh

**Affiliations:** Department of Orthopaedics Paraplegia and Rehabilitation, Pt B D Sharma PGIMS, 11-J/2-UH Medical Enclave, Rohtak, 124001 Haryana India

**Keywords:** Ankle fusion, Distal tibial tumours, Centralisation of fibula, Limb salvage surgery

## Abstract

**Background:**

Management of distal tibial tumours with limb salvage surgery poses a challenge for the orthopaedic surgeon. This study was done to evaluate the results of fibular centralisation as a technique to reconstruct defects that occurred after resection at this site.

**Materials and methods:**

Nine patients with a mean age of 23.2 years (range 17–34) with diagnosis of osteosarcoma in four patients, Ewing’s sarcoma in two, giant cell tumour in two and chondrosarcoma in one patient underwent surgical treatment for tumour in the distal tibia. All patients had wide resection of the tumour and ankle arthrodesis with centralisation of the fibula. Patients were assessed clinico-radiologically for bone union, infection and complications. The final functional outcome was estimated according to Musculoskeletal Tumor Society (MSTS) scores.

**Results:**

The mean age at the time of surgery was 23.2 years (17–34). There were five females and four males. The mean follow-up was 37 months (range 28–54 months). One of the patients with osteosarcoma had a recurrence a year after limb salvage surgery, underwent above-knee amputation, and died 18 months later due to metastasis. One patient developed leg length discrepancy. The mean MSTS score was 22.75 (range 17–27).

**Conclusion:**

Fibular centralisation is a durable reconstruction tool for defects of the distal tibial metaphysis with an acceptable functional outcome. It is an inexpensive and simple procedure, with a low rate of late complications, and reproducible results.

**Level of evidence:**

IV Retrospective case series.

## Introduction

Most bone tumours occur in the metaphysis of long bones. The standard protocol for treating malignant and locally aggressive tumours at this site is wide resection with which joint salvage is usually not feasible. Megaprosthesis reconstruction of the distal tibia has been proposed as an alternative in such a situation [1–3]. Though the results of megaprosthesis reconstruction have been reported to be good to excellent at other sites, it has not been as successful in distal tibial tumours [4]. When the ankle is involved, limb salvage presents unique difficulties both in terms of biomechanics and soft-tissue coverage. A variety of procedures which include free vascularised or non-vascularised fibula autograft and arthrodesis, osteoarticular allograft, Ilizarov technique and endoprosthesis [4–14] have been advocated for the reconstruction of segmental skeletal defects in this area and each of them have their own set of merits and demerits.

According to Casadei et al. [8] ankle arthrodesis is one of the best reconstructive procedures for limb salvage for distal tibial tumours. Though fibular centralisation has been well documented in cases of post-traumatic and post-infective tibial defects, osteomyelitis and congenital deformity [15–18], only a few series have documented use of fibular centralisation or the ‘fibula-pro-tibia’ procedure for reconstruction after excision of a tibial tumour [11, 12, 19]. In this procedure, the fibula is transferred to the tibia as a pedicle graft. As there is always a free blood supply to one end of the transplant the graft takes easily and frequently hypertrophies upon weight bearing over a period of time. [16, 17]. We report the clinical and functional outcome of excision of distal tibial tumours and limb salvage with ankle arthrodesis using fibular centralisation.

## Materials and methods

We retrospectively reviewed the results of nine consecutive patients with primary malignant and aggressive bone tumours of the distal tibia, which were treated by resection of the distal tibia and reconstruction of the defect by centralisation of ipsilateral fibula and ankle fusion between January 2005 and December 2009. Patients with involvement of the posterior tibial neurovascular bundle, fibula, ankle, tibio-fibular syndesmosis, pathological fracture and pulmonary metastasis were excluded from the study. Only those patients in whom the preoperative imaging was suggestive that satisfactory surgical margins could be achieved without resection of the ipsilateral fibula were included in the study. There were a total of five females and four males with a mean age of 23.2 years (range 17–34 years) and a mean follow-up of 37 months (range 28–54 months). All patients underwent detailed clinical examination, plain radiographs of the involved extremity and chest, MRI of the limb, CECT chest and whole-body isotope bone scan. A pre-operative biopsy was done after imaging studies so as to obtain a histopathological diagnosis. Staging studies were performed as per the Enneking staging system. As per our institution protocol, patients with sarcoma received neoadjuvant and adjuvant chemotherapy. MRI was used to define the extent of the lesion, the involvement of the soft tissues, its relation to the neurovascular bundle and the level of involvement of the bone. The diagnosis was osteosarcoma in four patients, Ewing’s sarcoma in two, giant cell tumour in two and chondrosarcoma in one patient.

The lesions were approached by way of an anterolateral incision, the exact position of which was determined by the site of biopsy and the location and extent of the tumour. Previous biopsy tracts were incorporated into the incision and were completely excised, meticulous dissection was carried out and an intra-articular distal tibia resection was done with a wide protective margin. In all the procedures, the level of the tibia resection was based on the proximal extent of tumour as determined by MRI. The bone was sectioned 2.5 cm above the upper margin of the tumour. Marrow from the remaining proximal tibia was sent for frozen-section evaluation. All articular cartilage was denuded from the upper dome of talus and a notch was created. An appropriate length of ipsilateral fibula with its retained peroneal muscle attachment was osteotomised through the same incision. In two cases we gave an extra small lateral incision at the distal end overlying the tip of the lateral malleolus, to detach the distal fibular ligaments The fibula was left about 2 cm longer than the defect and was mobilised medially so that the proximal end could be fitted into the medullary canal of the tibia. The distal end of the pedicled fibular graft was centralised to fit into the talar notch. Routinely, the proximal graft host required no fixation but a transcortical screw was used in two cases where the fibula did not fit snugly into the intramedullary canal. The distal end of the fibula was fitted into the talar dome and the junction was secured with two thick Kirschner (K) wires. The position of the fibula in relation to the tibia or ankle was adjusted and tibiotalar articulation was aligned. Care was taken to place the foot in 5°–10° valgus, 10° external rotation and plantigrade position. The mean length of resected bone was 11 cm (range 9–14 cm) and the mean length of fibula harvested was 13.5 cm (range 10–16 cm).

Soft tissue and skin were sutured and a well-padded plaster cast was applied from groin to toe, with the knee in 5º–10º flexion. Weight bearing was not allowed in the first 8 weeks. Stitches were removed after 3 weeks and the cast was reapplied. Guarded weight bearing was started from 8 to 10 weeks onward when radiological union began, and the full leg cast was converted to a below-leg cast after 16–20 weeks, when radiographs showed signs of boney union. Patients were assessed clinico-radiologically for bone union, infection and complications like nonunion of graft-host junction, implant failure, stress fracture of fibula and recurrence or metastasis at regular follow-ups. Functional assessment was done using the Musculoskeletal Tumour Society (MSTS) score [20].

## Results

As far as oncological results are concerned, all resected margins were histologically free of disease on intra-operative frozen sections and final analysis. One of the patients with osteosarcoma had a recurrence a year after limb salvage surgery, underwent above-knee amputation and died 18 months later due to metastasis, leaving a total of eight patients for final outcome analysis. The mean MSTS score at the time of final follow-up was 22.75 (range 17–27), and all patients were able to perform their routine work, though with some restriction in running and climbing stairs (Table 1).

**Table 1:** Clinical details of the patients

Sr. no	Age	Sex	Diagnosis	Complication	Additional procedure	Leg length discrepancy (cm)	Time to FWB (months)	MSTS Score	Follow-up (months)
1	22	M	Osteosarcoma	–	–	–	8.5	25	54
2	19	F	GCT	Nonunion proximal graft host junction	Secondary bone grafting	1.5	19	23	41
3	20	M	Osteosarcoma	Nonunion at arthrodesis site	Secondary bone grafting	2	18	17	38
4	34	F	Chondrosarcoma	–		–	7	22	36
5	23	F	Ewing sarcoma	–		1.5	12	19	36
6	20	M	Osteosarcoma	Local recurrence	Amputation	–		Died due to lung metastasis at 18 months	
7	30	F	GCT	–		–	12.5	23	32
8	24	F	Ewing sarcoma	–		–	14	27	31
9	17	M	Osteosarcoma	Limb length discrepancy		7	8	26	28
Mean	23.22					1.33	12.4	22.75	37
±SD	±5.49					±2.27	±4.49	±3.41	±8.02

*M* male, *F* female, *GCT* giant cell tumour, *FWB* full weight bearing

**Fig. 1:**
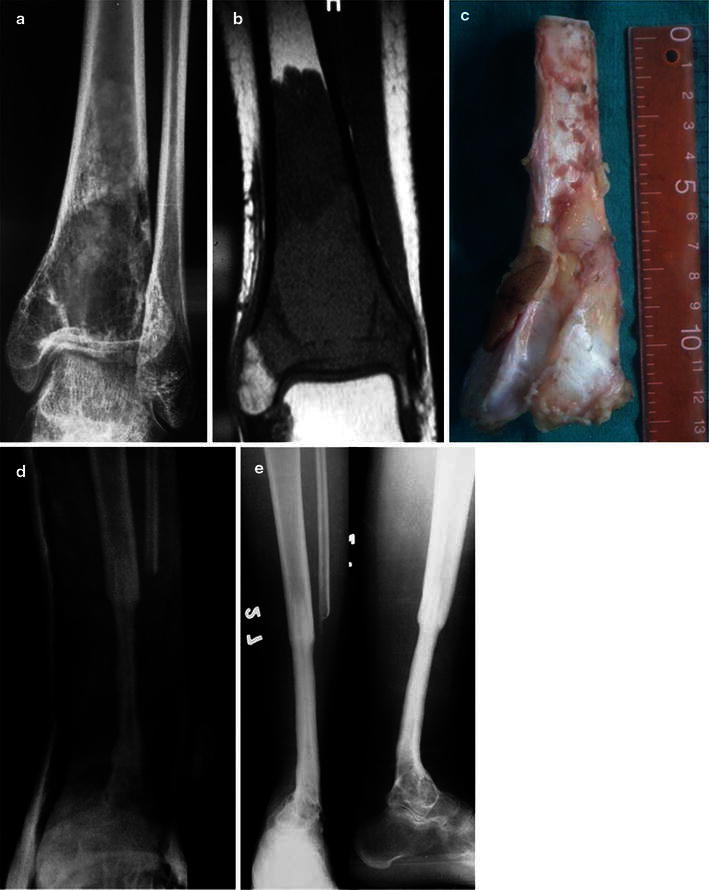
**a** Radiograph showing osteosarcoma of the distal tibia in a 22-year-old male, **b** MRI of the same patient, **c** resected distal tibia, **d** X-ray at 8.5 months follow-up, **e** X-rays at 54 months follow-up showing hypertrophy of the fibula

**Fig. 2:**
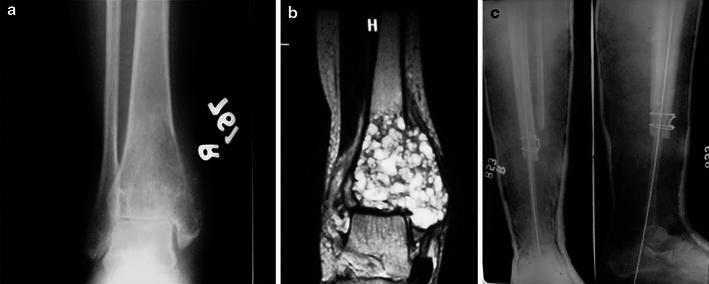
**a** Radiograph showing giant cell tumour in a 30-year-old female, **b** MRI of the same patient, **c** post-operative radiographs

Non-oncological results were also satisfactory. Full and unprotected weight-bearing on the operated leg was achieved at an average time of 12.4 months (range 7–19 months). Radiographically, hypertrophy of the grafted fibula was observed in all the patients (Fig. 1). One patient had nonunion at the proximal graft-host junction; cancellous bone grafting of the nonunion site was done (graft harvested from ipsilateral iliac crest), along with K-wire fixation 9 months after the index surgery. Union was achieved and the patient was doing unprotected full weight bearing after another 10 months. Primary union of the ankle occurred in all but one of the patients, supplementary cancellous bone-grafting was done 10 months after the index surgery and the limb was immobilised in cast for another 6 months. Union was achieved and unprotected full weight bearing walking was initiated after another 4 months. There was no superficial or deep wound infection. One skeletally immature patient whose distal tibial epiphysis was resected developed a leg-length discrepancy of 7 cm and was advised to use a shoe raise. Though there was no case of stress fracture of the fibula, a slight angulation at the junction of the proximal end of the transferred fibula was seen in one case. The degree of angulation was minor and did not require surgical intervention (Fig. 2).

## Discussion

In the past, below-knee amputation used to be the standard surgical treatment for distal tibial tumours and limb salvage surgery was rarely considered. With advances in neoadjuvant chemotherapy and oncological treatment, various limb salvage reconstructive techniques have come up which include large segmental allografts, vascularised or avascular autografts, osteoarticular allograft, bone transport, endoprosthesis and reimplantation of the tumour-bearing bone segment after the devitalization of the tumour cells by heating, freezing, or extracorporeal irradiation [4–14]. However, reconstructing defects after resection of a distal tibial tumour remains challenging due to precarious soft tissue cover at this site. When evaluating a reconstruction technique, the factors which need to be considered are the ease of the procedure, its morbidity, complications, functional outcome and durability. The use of the avascular strut allograft is often limited by the available length of the resection, risk of nonunion, fracture and infection, besides the fear of disease transmission [21]. The use of a free-vascularised fibular transfer from the contralateral limb adds morbidity to the normal limb, is time-consuming and requires a specialised microsurgical team. Free fibular graft from the contralateral leg in the presence of extensive dissections may necrose; and even if it survives it may take a long time to heal with poor functional outcome [15]. Distraction osteogenesis using the Ilizarov technique is a time consuming procedure. Although early weight-bearing is possible, there is risk of pin track infection and the frame is not very well accepted by most of the patients [22]. Endoprosthetic replacement of the distal tibia has the potential advantage of immediate stability and rapid restoration of ankle function. However, there is paucity of data on long-term evaluation of use of megaprostheses of the ankle, and the short-term results are not encouraging. It has been reported to be associated with high failure rates related to the instability, malalignment or difficulties in anchoring the prosthesis to the talus. Other reported complications include infection, inadequate soft tissue coverage and talar collapse [4, 23]. Abudu et al. [4] in their series of endoprosthetic replacement reported rapid deterioration in a patient’s ultimate outcome despite good initial functioning. Natarajan et al. reported a secondary amputation rate as high as 50 % after limb salvage with megaprosthesis. In their study of six patients with prosthetic replacement, four required soft tissue reconstruction with a muscle pedicle flap. Of the other two patients, one developed flap necrosis and deep infection and finally underwent amputation. Two patients developed local recurrence of the tumour and also required above-knee amputation [24].

**Fig. 3:**
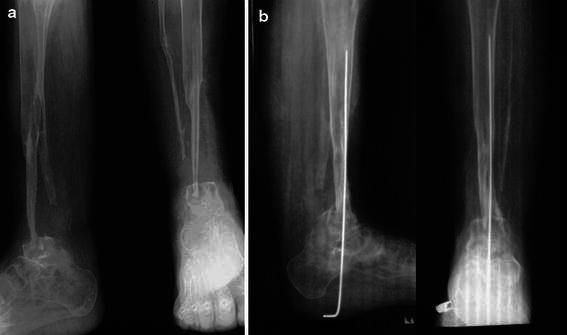
**a** Radiographs of a 20-year-old male, showing nonunion at the arthrodesis site. A streak of new bone formation from the remnant periostium can be seen at the site from which the fibla was harvested. **b** Secondary bone grafting was done, along with long K-wire fixation: radiographs at final follow-up showing union at the arthrodesis site along with hypertrophy of the fibula

Arthrodesis has been regarded as one of the best options for skeletal reconstruction after bone tumour resection of the distal tibia and ankle. Casadei et al. [8] reported good functional and oncological results in 12 patients with malignant bone tumours of the distal tibia treated by resection and arthrodesis using an autogenous bone graft. Bishop et al. [6] too reported satisfactory results using vascularised bone graft for reconstruction of segmental bone loss. The advantages of an arthrodesis are that it restores skeletal continuity, provides excellent stability and avoids problems related to prosthetic implantation. Centralisation of the fibula is usually performed for post-traumatic and post-infective tibial defects but only a few studies have evaluated its role in reconstruction of tumour-related defects [11, 14, 25]. Ipsilateral fibular transfer is an easy, simple, inexpensive biological procedure that does not require micro-vascular skills. The autogenous fibular graft has some important advantages over other donor sites because of its length, geometrical shape and mechanical strength. The fibula being a long, straight tubular bone with predictable anatomy and shape allows tibial intramedullary insertion (Fig. 3). A large graft of the ipsilateral fibula raised on a pedicle of peroneal artery, aligned and fixed to the tibia in its posterior long axis, provides a sound mechanical and biological basis for union [26]. The fibula is surrounded by muscles all around and has abundant vascular supply from the nutrient branch of the peroneal artery and circular anastomosis of the musculoperiosteal vessels, which supports it in its hypertrophy and union at the synostotic site [26]. The reduction in volume of the lower leg that follows antero-medial shift of the fibula makes skin closure easier, even in cases with loss of soft tissue. The shorter operating time and the fact that the graft retains its blood supply may help to reduce infection, improves its chances of union and accelerates the process of hypertrophy [11, 27].

When the fibula is subjected to more than normal weight bearing stresses, it undergoes hypertrophy and becomes an integral part of static supporting architecture of the leg. Hypertrophy occurs more commonly when the limb is mechanically loaded [28, 29]. Our rehabilitation schedule did not depend on hypertrophy of the fibula; patients progressed to full weight-bearing once there was radiological evidence of bony union. Hypertrophy was seen later after continued weight bearing. The morbidity of this procedure was low regarding frequency and type of re-operations. We were able to obtain adequate margins with primary closure of the skin in all our cases and did not encounter complications like wound dehiscence, deep infections and skin sloughing in any of our patients. We used minimal implants in the present study (K-wires in all cases and screws in two cases). Hardware increases the risk of infection. Implant exposure secondary to skin necrosis (especially after chemotherapy), and painful hardware are the most common causes of reoperation [29].

The two disadvantages of this procedure are loss of movement at the ankle joint and a little leg length discrepancy in skeletally immature patients. Fortunately, distal epiphysis is not a major contributor to limb length, and the discrepancy is less than that seen around the knee. Moreover, they were well tolerated by our patients, without major disabilities. We encountered no donor site morbidity like motor weakness and flexion contracture of the toes, as muscles originating from the transferred fibula were left unreleased.

The reconstruction of a large defect resulting from resection of a tumour has always been difficult. Arthrodesis with centralisation of the fibula is a relatively straightforward procedure, requiring no microsurgical expertise, giving durable and satisfactory functional results.
